# Enhancement of Cadmium Phytoremediation Potential of *Helianthus annuus* L. with Application of EDTA and IAA

**DOI:** 10.3390/metabo12111049

**Published:** 2022-10-31

**Authors:** Naila Shah, Muhammad Qadir, Muhammad Irshad, Anwar Hussain, Muhammad Hamayun, Waheed Murad, Ajmal Khan, Ahmed Al-Harrasi

**Affiliations:** 1 Department of Botany, Garden Campus, Abdul Wali Khan University Mardan, Mardan 23200, Pakistan; 2 Department of Botany, Government Girls College, Mardan 23200, Pakistan; 3 Natural and Medical Sciences Research Center, University of Nizwa, P.O. Box 33, Birkat Al-Mauz, Nizwa 616, Oman

**Keywords:** sunflower, EDTA, IAA, short exposure duration, efficient cadmium remediation

## Abstract

The aim of the current study was to assess the cadmium (Cd) phytoremediation potential of *Helianthus annuus* L. that was exposed to 50, 100, and 150 mg/kg of cadmium for 15, 30, and 60 days with application of EDTA (Ethylenediaminetetraacetic acid) in the soil and IAA (indole acetic acid) as a foliar spray. The results indicated that the concentration, duration of exposure, and amount of Cd affect the phytoremediation potential. The maximum Cd was observed at 60 days (32.05, 16.86, and 10.63%) of Cd application, compared to 15 (2.04, 0.60, and 1.17%) or 30 days (8.41, 3.93, and 4.20%, respectively), in a dose-dependent manner. The application of EDTA in the soil and foliar IAA enhanced the Cd accumulation in the plants at 15, 30, and 60 days of exposure, with maximum accumulation at 60 days. Exposed plants with foliar IAA application showed 64.82%, 33.77%, and 25.84% absorption at 50, 100, and 150 mg/kg, respectively. Apart from higher absorption, the cadmium translocation to the edible part of the plants ceased, i.e., the seeds had 0% accumulation. The interesting fact was recorded that efficient phytoremediation was recorded at 15 days of exposure, whereas maximum phytoremediation was recorded at 60 days of exposure. To minimize the stress, the host also produced stress-related metabolites (i.e., flavonoids, phenolics, proline, and sugar) and antioxidants (i.e., catalases and ascorbate peroxidases). From the current evidence, it could be assumed that the use of EDTA and IAA, along with hyperaccumulating plants, could be a possible green method to remediate Cd-contaminated soil efficiently in a short period of time.

## 1. Introduction

The release of heavy metals to the environment is very common in the current world. They mainly contaminate the soil and water, which plays havoc with the lives of both flora and fauna [1]. Soil reclamation through physical means is a cost-effective, labor-consuming, and time-efficient process with many side effects in the form of environmental imbalance in nutrients uptake, disturbed food chains, and effects on various nutrient-cycling phenomenon [2]. Moreover, increasing demand for food and forage crops for the world’s population is the need of the hour. Striving for the fulfillment of the need for food for human beings and livestock results in the contamination of the environment by various agencies [3]. Anthropogenic activities are the main forces responsible for the contamination of soil by heavy metals [4]. The toxicity levels of heavy metals increase with increases in their sources and decrease in sinks or with no sinks. In such a scenario, even an essential heavy metal becomes toxic [5].

Cadmium is one of the heavy metals that is nonessential, and beyond the threshold levels is highly toxic and lethal to micro- and macro-flora and -fauna. The total Cd levels present in the soil do not inevitably reflect the Cd bioavailability to the host plants [6] because Cd’s varied distributions (adsorbed vs. free) and various chemical configurations (chemical speciation) impact its phytoavailability. Because it is primarily present in soil attached to the exchangeable solid phases, cadmium is comparatively accessible for plant absorption [7] and thus readily released into the soil solution. Cd is mostly found in the soil solution as the Cd2+ ion or as inorganic or organic compounds. Cd may reversibly bind to soil particles such as organic matter or Fe and Mn oxides in the solid phase. Cd is mostly absorbed by plants when they come into contact with pore water, which is the end result of Cd being divided between the liquid and solid phases of soil [8]. Damage to agricultural crops is a serious matter for plant physiologists and ecologists as a result of Cd availability and uptake [9]. It results in a reduction in the yield of the crop and, on the other hand, acts as a serious pollutant for soil and water. Apart from that, the cadmium is considered a group A contaminant and has carcinogenic and mutagenic properties in plants, animals, and particularly humans [10].

Due to ever-growing populations and increasing food demands, these contaminated lands can be left barren. In developing countries such as Pakistan, the cadmium concentration ranges from 0.03 to 0.07 mg/L [11]. To decrease the gap between production and demands, these agricultural lands must be made cultivable and contaminant-free. To remove the contaminants, particularly Cd, from the environment, several techniques are in practice to identify a cost-effective and viable method among them. Phytoremediation is considered a green way to remove the metal from the environment. It is a “plant-based method”, in which elemental contaminants are extracted and removed from the environment or have their soil bioavailability reduced [12,13]. Even at low quantities, ionic substances in the soil can be absorbed by plants through their root systems. In order to absorb heavy metals and control their bioavailability, plants stretch their root systems into the soil matrix and create rhizosphere ecosystems, recovering the contaminated soil and maintaining soil fertility [14]. The differential adsorption–desorption properties of heavy metals alone and in combinations have differential effects on their transport in the soil, which may be utilized efficiently in phytoremediation practices. For instance, the recovery ratio of Cd ions from porous matter was higher than Pb ions, which was attributed to the lower adsorption of Cd ions on the solid matrix [15]. Similarly, the transport of Pb was found to be influenced by silica powders in porous media [16].

The use of phytoremediation has benefits. In addition to being easy to manage and inexpensive to install and maintain, phytoremediation has the following advantages: (i) it can reduce the introduction of pollutants to the environment and ecosystem; (ii) it can be used on a large-scale field; (iii) it can be easily disposed of; (iv) it prevents erosion and metal leaching by stabilizing heavy metals, reducing the risk of contaminants spreading; and (v) it stabilizes heavy metals [17]. In the current study, sunflowers were used as remedial plants for cadmium remediation, whereas EDTA was supplemented in the soil and IAA was applied as foliar spray to enhance their remediation potential and efficiently remove the cadmium from the contaminated site.

## 2. Materials and Methods

### 2.1. Soil Preparation

Plant growth medium was prepared by mixing sand (~0.5 mm), clay (~0.002 mm), and manure in a 2:1:1 ratio to make a sandy loam for better sunflower growth. The pots were filled with 5 kg of soil and were kept in the screen house at Abdul Wali Khan University Mardan.

### 2.2. Experimental Design

For the pot experiment, healthy and viable seeds of *H. annuus* L. (Hysun-33) were obtained from the agriculture research center in Mardan. The seeds were surface-sterilized with 70% ethanol, followed by rinsing with sterilized distilled water to remove the ethanol. The experiment was a factorial combination of three factors, i.e., Cd treatments (0, 50, 100, and 150 mg/kg of soil), EDTA supplemented in the soil in the form of an EDTA solution (5 mM (500 mL of solution in each pot)), the foliar application of IAA using a portable spray machine (2.5 µM solution sprayed at 5-day intervals until final harvest), and the combination of EDTA and IAA with selected metal levels. All the treatments had three replicates with four plants per replicate, which were allowed to grow in greenhouse conditions. The pots were properly irrigated with common tap water systematically in the morning and evening. For plants harvested at 15 and 30 days of exposure, only the root shoot lengths, fresh and dry weights, and metal contents were recorded. However, for plants harvested after 60 days of exposure, the effects of cadmium, EDTA, and IAA were recorded on different parameters, including growth and physiological attributes and cadmium accumulation in the presence and absence of EDTA and IAA.

### 2.3. Estimation of Indole Acetic Acid and Salicylic Acid

The indole acetic acid was determined by the protocol used by Hussain and Hasnain [18], whereas the salicylic acid was estimated using the protocol of Warrier et al. [19].

### 2.4. Quantification of Metabolites

The total flavonoid contents were extracted by macerating 0.5 g of fresh leaves of the host in 5 mL of 80% ethanol and incubating for 24 h in a shaker. Following incubation, centrifugation was performed at 10,000 rpm at 25 °C for 15 min. Pellets were removed, and supernatants were used for flavonoid determination using the AlCl_3_ method, as mentioned earlier [20].

To extract the phenolics, 1 g of plant leaves were crushed in 16 mL of ethanol and incubated at 30 °C for 3 h. Following incubation, centrifugation was performed for 10 min at 10,000 rpm in normal conditions. The supernatants were filtered via Whatman No. 42 filter paper, and the volume was reduced to 1 mL using a rotary evaporator at 40 °C. The concentrated filtrate was resuspended in 10 mL of dH_2_O, and phenolics were determined using the method of El Far [20].

The extraction of proline was performed from the leaves of the host using 0.2 g of fresh leaves (macerated to fine powder in liquid nitrogen) with 1 mL of 60% ethanol. The resultant mixture was kept for incubation at 4 °C for about 24 h. The reaction mixtures were centrifuged for 5 min at 10,000 rpm. To remove nearly all of the proline from the leaves, the procedure was repeated. Proline was estimated using the technique of Bates et al. [21].

### 2.5. Determination of Antioxidant Response

To assess the CAT activity, the cleavage of H_2_O_2_ was assessed [22]. A mixture of 3% H_2_O_2_ (0.4 mL) and 0.1 mM EDTA in 2.6 mL of 50 mM PBS (pH 7) was added to 0.1 mL of supernatant. The decrease in H_2_O_2_ was noted by the decline in optical density at 240 nm, which was considered degradation by μM H_2_O_2_ min^−1^.

The protocol of Asada [23] was used for the estimation of APX in the leaves of the host. For the reaction to start, approximately 0.2 mL of leaf extracts were mixed with 0.1 mL of 0.5 mM ascorbic acid, 0.6 mL of 50 mM PBS (pH 7.0), and 0.1 mL of 0.1 mM H_2_O_2_. The decline in O.D was noted at 290 nm and expressed as U mg^−1^ protein (U = change of 0.1 absorbance min^−1^ mg^−1^ of protein).

The protein contents were estimated for each extract according to Bradford [24]. The chlorophyll and carotenoid pigments were quantified according to the method reported by Schoefs [25].

### 2.6. Estimation of the Metal in Plant Biomass

For the estimation of the metals in the plant parts treated with the aforementioned levels of Cd, 0.5 g of oven-dried samples were weighted and subjected to acid digestion. The process of acid digestion was started by adding 1 mL of perchloric acid (HClO_4_) and 4 mL of nitric acid (HNO_3_) to the oven-dried plant samples. The mixture was filtered using Whatman 42 filter paper after cooling at 30 °C. With distilled water, the mixture’s final volume was changed to 25 mL. As a positive control solution, control plant samples underwent the same processing as the experimental samples. Similar steps were taken to produce the blank solution but without including the sample. The cadmium was quantified through an atomic absorption spectrophotometer (Perkin–Elmer model 700, MA, USA) following the protocol of Amin et al. [26].

### 2.7. Data Analysis

The experiments were repeated three times, and the data obtained from the factorial experiments were grouped into cadmium, cadmium/EDTA, cadmium/IAA, and cadmium/EDTA/IAA treatment conditions. An analysis of variance and Duncan’s multiple range test were performed using SPSS Statistical Package v. 21 (IBM, Armonk, NY, USA) to determine the significance at *p* ≤ 0.05.

## 3. Results

### 3.1. Effects of Cadmium, EDTA, and IAA on the Agronomic Attributes of H. annuus L.

**Figure 1:**
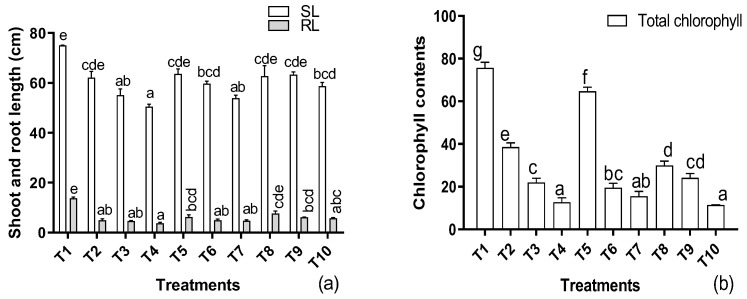
Effects of different levels of Cd under EDTA and IAA application on root and shoot length (**a**) and chlorophyll contents (**b**) of *H. annuus.* Data are the means of three replicates ± standard errors, where the different letters represent significant differences at *p* ≤ 0.05. T1 = control, T2 = 50 mg/kg Cd, T3 = 100 mg/kg Cd, T4 = 150 mg/kg Cd, T5 = 50 mg/kg Cd + EDTA, T6 = 100 mg/kg Cd + EDTA, T7 = 150 mg/kg Cd + EDTA, T8 = 50 mg/kg Cd + EDTA + IAA, T9 = 100 mg/kg Cd + EDTA + IAA, T10 = 150 mg/kg Cd + EDTA + IAA.

When exposing sunflowers to the aforementioned supplementation of Cd and the application of the EDTA and IAA, the growth attributes were influenced significantly (Figure 1a). Dose-dependent decreases of 15, 22, and 25% were recorded at 50 mg/kg to 150 mg/kg, respectively, in the shoot and root length of the host plants. The application of EDTA in the presence of Cd significantly improved the shoot and root length as the level of Cd increased. However, the length was lower compared to the untreated control. On the other hand, a similar enhancement in the shoot and root length was also recorded upon the foliar application of IAA, reducing the effects of Cd by 8, 9, and 7% at 50, 100, and 150 mg/kg, respectively. Similar declines were also noted in the case of the total chlorophyll contents of the host plants, showing dose-dependent declines as the metal concentration increased from 0 mg to 150 mg/kg (Figure 1b). An improvement was recorded with the application of EDTA with the application of 50 mg/kg of Cd. However, no further improvement was recorded with the application of EDTA or IAA, separately or in combination, at all concentrations of the metal in the soil.

### 3.2. Determination of Phytohormones

**Figure 2:**
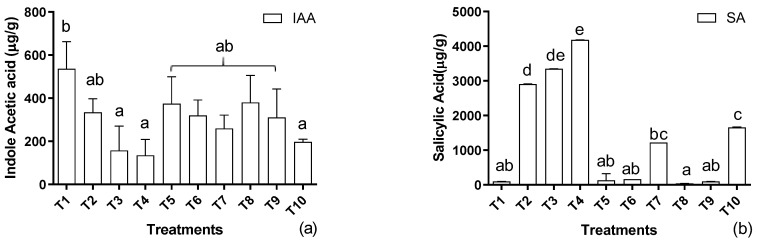
Effects of Cd levels, EDTA, and IAA on (**a**) indole acetic acid and (**b**) salicylic acid of *H. annuus.* Data are the means of three replicates ± standard errors, and the letters represent significant differences (*p* < 0.05).

During the exposure of the host plant to the aforementioned levels of cadmium, a concentration-based significant decline was noted in the endogenous IAA synthesis in the sunflower as the metal level increased in the soil (Figure 2a). The supplementation of EDTA in the soil and IAA as a foliar spray improved the IAA production. However, the amount of endogenous IAA was lower than that of untreated plants. A contrary tendency was noted in the case of SA production, showing an opposite trend to IAA production (Figure 2b). A direct relation of SA with the metal concentration was recorded, where an increase in the metal concentration increased the SA production. However, the application of EDTA and IAA inversely regulated the SA production, i.e., with the application of EDTA and IAA and an increasing metal concentration, a decline was recorded in the salicylic acid production.

### 3.3. Determination of Metabolites

**Figure 3:**
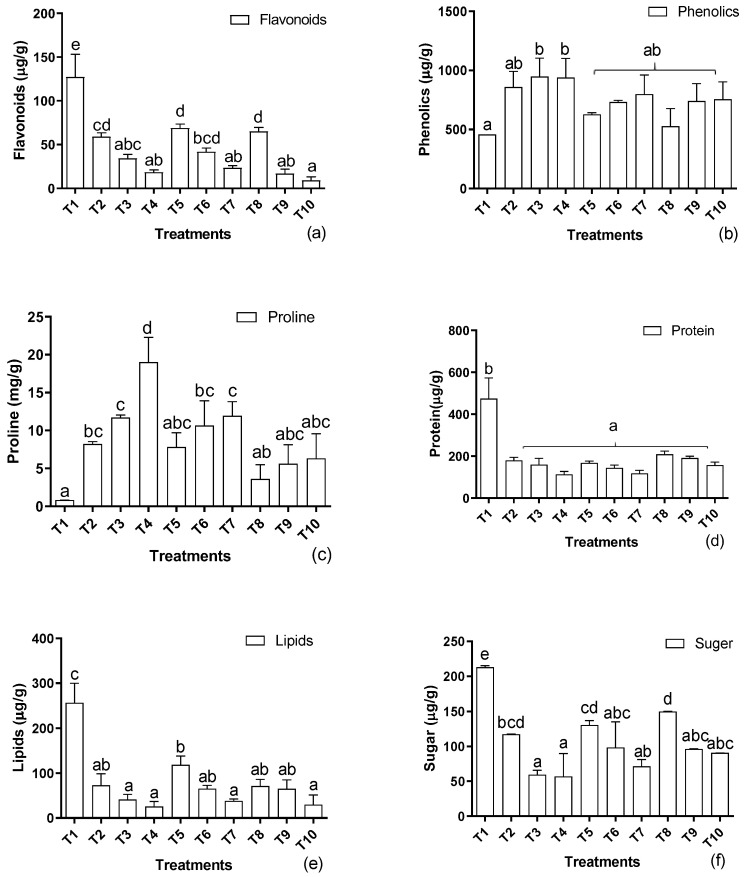
Effects of Cd levels, EDTA, and IAA on (**a**) flavonoids, (**b**) phenolics, (**c**) proline, (**d**) protein, (**e**) lipids, and (**f**) sugar contents of *H. annuus.* Data are the means of three replicates ± standard errors, and the letters represent significant differences (*p* < 0.05).

Treating plants with the selected levels of cadmium negatively impacted the endogenous flavonoid production in the host plants. A concentration-based reduction in the accumulated flavonoid content was recorded (Figure 3a). The treatments of EDTA in the soil and IAA as a foliar spray significantly improved the flavonoid production of the host. However, the amounts were lower in comparison to the untreated control plants. On the other hand, endogenous phenolics were boosted when the soil was supplemented with the mentioned concentration of cadmium and showed a positive correlation with cadmium levels (Figure 3b). Similarly, the application of EDTA and IAA further significantly improved the phenolic production in comparison to the untreated hosts. A similar tendency was also noted in the case of endogenous proline production, i.e., increases in the cadmium levels were associated with accumulated proline contents, and they showed a direct proportion (Figure 3c). Improvements were also recorded with the application of EDTA and IAA in the presence of the mentioned supplements of cadmium in the soil.

Significant declines in the total protein and lipid levels were recorded in the host plants when the aforementioned levels of cadmium were supplemented in the soil (Figure 3d). EDTA-amended soil and foliar IAA enhanced the protein and lipid contents. However, the quantity was lowest compared to the untreated plants (Figure 3e). Similar tendencies were also noted in the accumulation of the total sugar contents of the sunflowers, which showed a decline with the increase in cadmium concentration (Figure 3f). Significant improvements were recorded with EDTA and IAA application. However, the sugar contents remained lower than in the control plants.

### 3.4. Antioxidant Response

**Figure 4:**
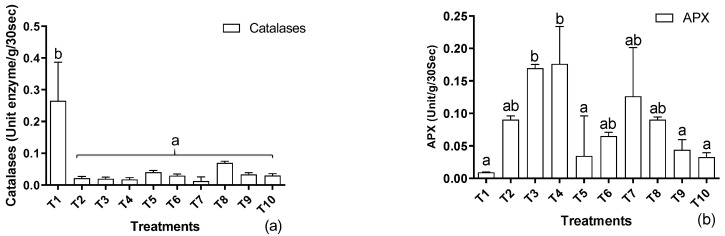
Effects of Cd levels, EDTA, and IAA on (**a**) catalases and (**b**) ascorbate peroxidase activity of *H. annuus.* Data are the means of three replicates ± standard errors, and the letters represent significant differences (*p* < 0.05).

Plants in stress boost their antioxidant system to cope with the primary and secondary stressful conditions. In the current scenario, treating plants with cadmium decreased the production of catalases as the metal level increased in the soil (Figure 4a). No further improvements were recorded with the application of EDTA in the soil or the foliar application of IAA at all supplemented levels of cadmium. A contrasting tendency was noted in the case of ascorbate peroxidases, indicating a multifold increase with the increase in metal in the soil up to 150 mg/kg (Figure 4b). Similar improvements were also recorded with the application of EDTA and IAA. Nonetheless, the enzyme units were lower than in cadmium-stressed plants.

### 3.5. Metal Determination

**Figure 5:**
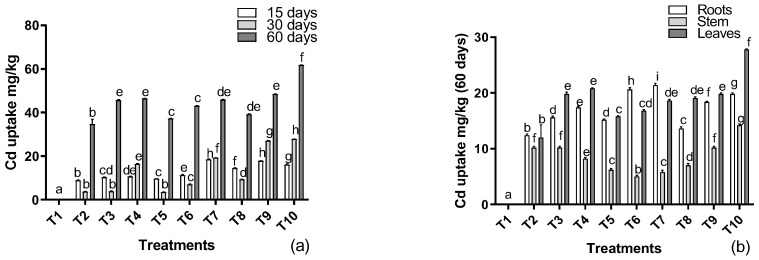
Effects of Cd levels, EDTA, and IAA on (**a**) cadmium uptake and (**b**) translocation to the aerial parts of *H. annuus.* Data are the means of three replicates ± standard errors, and the letters represent significant differences (*p* < 0.05).

The total cadmium contents in the plant parts increased with the increase in cadmium supplementation in the soil from 0 to 150 mg/kg (Figure 5a). A similar tendency was also noted in the case of the application of EDTA, showing increased accumulation with the increase in metal in the soil. In the case of the foliar application of IAA, an even higher accumulation was recorded as the cadmium supplementation was elevated in the soil. The highest accumulation was noted in the plants treated with the foliar application of IAA, followed by EDTA application and only cadmium-stressed plants. Similarly, the accumulation was enhanced by an increase in exposure time. A lower accumulation was recorded in the plants exposed for 15 days to cadmium supplements. An interesting result was found in case of 30 days of exposure, which showed a decline in accumulation compared to 15 days of exposure. However, after 60 days of exposure, the maximum increase was recorded in the accumulation of cadmium from the soil, showing a direct relation to the duration of exposure to metals supplemented in the soil.

Similar patterns were also recorded in the case of translocation of the metal to the aerial parts of the plants (Figure 5b). A higher amount was recorded in the roots of the plants, followed by the leaves of the host. The lowest accumulation was recorded in the stem, and no accumulation was recorded in the seeds of the sunflowers. When treating plants with cadmium levels, an increase in the accumulation in the roots was recorded. However, the application of EDTA in the soil and foliar IAA further enhanced the accumulation in the roots, thereby enhancing phytoremediation potential. A contrasting tendency was noted in case of the stems, i.e., with the inclination of metal level, the accumulation decreased in a concentration-based manner. The application of EDTA in the soil induced a significant improvement in the translocation to the stem. Interesting results were recorded when foliar IAA was applied, showing the highest accumulation in the stems at higher levels of cadmium supplementation. In the case of the leaves, a dose-dependent increase was recorded with the cadmium levels. Nonetheless, EDTA application lowered the translocation of cadmium to the leaves in a concentration-based manner in comparison to the stressed control plants. Similarly, a significant increase was recorded with IAA application, showing higher translocation and the accumulation of cadmium in the leaves.

## 4. Discussion

The increase in global population requires higher yields and production, which could be possible with better soil health and balanced nutrient conditions. Soil reclamation and the removal of these toxic metals are challenging tasks for agriculturists and other plants scientists [27,28]. The removal of toxic heavy metals from the agroecological zones in a sustainable way is the call of the day [29,30,31]. Among the techniques, phytoremediation with hyperaccumulating plants with short lives could be a possible solution to maintain better health in soils and increase their productivity to meet the food demands for the ever-growing human populations [32]. In the current scenario, sunflowers supplemented with the aforementioned levels of the cadmium were severely impaired in terms of plant height, root length, and chlorophyll contents [33]. The chlorophyll content decreased in a dose-dependent manner in response to cadmium. A similar pattern of chlorophyll reduction has been reported in previous studies [34,35,36,37,38,39]. The excessive ROS production leads to the oxidation of the chlorophyll contents, leading to their degeneration and degradations as a result of secondary oxidative stress. The inhibition of chlorophyll may be attributed to the inhibition of enzymes involving pigment biosynthesis in response to cadmium, as has been previously demonstrated by Qian et al. (2009). Moreover, cadmium stress has shown to induce an imbalance in cell redox homeostasis, which leads to oxidative damage in plants (Hendrix et al., 2020).

The application of EDTA in the soil and foliar IAA reduced the effects of Cd by 8, 9, and 7% at 50, 100, and 150 mg/kg, respectively. To cope with stressful environmental constrains, plants produce a range of substances, including phytohormones, such as IAA and SA, and other metabolites, such as flavonoids, phenolics, proteins, proline, and sugar, to maintain homeostatic conditions inside the cell and maintain cell viability under environmental constrains [40,41]. Among the phytohormones, IAA not only acts as a plant growth promotor, but recently it was also revealed that it has a role in stress mitigation [42]. In the current scenario, a decline in the total IAA content was recorded with the increase in metal concentration in the medium. This was probably due to the fact that beyond a threshold level the metal causes the breaking down of IAA, which results in lower synthesis and the accumulation of IAA by the host, resulting in lower growth, development, and hence lower biomass production, which was recorded in the current study [43]. On the other hand, salicylic acid is a stress hormone that showed an increasing pattern with higher metal levels. Additionally, SA is known to reduce stress in individuals from several kingdoms, including humans, plants, and animals. Along these lines, it stands to reason that perhaps the host could be employing increased production as a stress management technique. In order to help their host to survive the harsh situation, chaperones, heat shock proteins, antioxidants, and genes involved in the manufacture of secondary metabolites such as sinapyl alcohol dehydrogenase, cinnamyl alcohol dehydrogenase, and cytochrome P450 are all activated by SA [44,45]. In the current condition, SA production was enhanced with increases in the metal concentration in a dose-dependent manner, which is an effective stress-mitigating strategy for ameliorating host stress tolerance [46]. Phytohormones and other stress-related metabolites relieve cadmium, and the resultant ROS accumulation also plays a key role in the mitigation of cadmium stress in sunflowers by improving growth and strengthening the antioxidant and metabolic systems [47]. To cope with metal stress, plants produce a range of secondary metabolites, including flavonoids, phenolics, low-molecular-weight protein, and sugar and proline contents [48]. In the current condition, lower flavonoid contents were recorded upon exposure to the mentioned levels of cadmium [49]. These lower flavonoids were perhaps because cadmium-induced stress results in the disturbance of the phenylalanine pathway, resulting in lower flavonoid production [50,51]. On the contrary, higher levels of phenolics were recorded at all mentioned levels of the metal. The phenolics were acting as effective ROS quenchers to relieve the stressful metal conditions [52]. Similarly, higher proline contents were recorded with the exposure of aforementioned concentrations of the metal. The proline contents act as an osmolyte, maintaining the host osmotic adjustment [53,54,55]. On the other hand, the protein, lipid, and sugar contents showed declines with the increase in metal [56]. This was probably due to the fact that a higher metal level caused toxicity to the cell, leading to systematic cell death. In some cases, the metal acts as a competing inhibitor and binds with the active sites of the enzymes, thus making them malfunction, resulting in lower production of protein, lipid, and sugar contents [57].

Plants produce ample amounts of antioxidants in response to environmental constrains, which aid the host to cope with the ROS produced as a result of the environmental stressors. In the current findings, lower catalase activity was recorded in all treatments compared to the control plants [58]. On the contrary, higher ascorbate peroxidase levels were recorded for each increase in metal in the soil. Higher ascorbate peroxidase contents showed efficient ROS scavenging and enhanced the stress tolerance of the host plants [59]. In order to control the ROS levels and preserve cellular homeostasis under stress, plants have a battery of antioxidant molecules. One essential antioxidant enzyme of such scavenging systems is ascorbate peroxidase (APX). It utilizes ascorbate as an electron donor to catalyze the transformation of H_2_O_2_ into H_2_O and O_2_. In response to environmental challenges and during typical plant growth and development, APX expression is variably regulated. Depending on their subcellular location and the presence of certain regulatory elements in the upstream regions of the corresponding genes, various isoforms of APX exhibit distinct responses to environmental stressors [60]. In a previous study, the supplementation of thiourea as a scavenger of ROS changed the expression of various arsenic (As)-related transporter genes in flag leaves and developing grains (inflorescence) of rice [61]. The antioxidant enzymes were also altered, and as accumulation was significantly reduced in rice. Moreover, vermicomposting in conjunction with phytoremediation can be an efficient strategy to restrict the bioavailability of soil pollutants [61].

Different factors affect the bioavailability of cadmium to the host plants, including pH, moisture, and the temperature of the surroundings. The bioavailability of cadmium increased with an increase in the pH based on pore water concentrations, explaining the reduced competition of H+ ions making cadmium more bioavailable in pore water at a high pH [62]. Cadmium sorption to the soils, estimated from water-soluble concentrations, was not significantly affected by the soil moisture content [63]. In a study with ryegrass, the uptake of both 109Cd and 65 Zn and their stable isotopes was higher in ryegrass grown at 21 °C than that grown at 9 °C. Results from a fractionation and speciation analysis of soil cadmium and zinc were correlated with plant uptake, and there was a good consistency between the observed plant uptake, the physicochemical forms of cadmium and zinc in the soil, and the soil solution presumed to be available to the plants [64]. However, in a study with metal and silicon, the bioavailability decreased with an increase in temperature [15]. The absorption of the metal by the host root showed a multifold dose-dependent increase when sunflower seedlings were grown in a soil condition spiked with the mentioned levels of the selected metal [45,65]. The higher uptake leads to higher bioremediation of the environment [48,66,67]. On the contrary, an increase was recorded in the Cd accumulation with the application of EDTA [68]. Similarly, the application of IAA tends to increase the accumulation of cadmium in the plant parts [69,70]. The IAA helps the plant to adjust to abiotic stresses. Moreover, IAA is an acidic hormone that helps to make the cell wall more flexible to expansion and cell division [71,72]. As a result of cell expansion and cell division, more compartments are available for metal compartmentalization, thereby dividing the stress to minimize its effects and allow the plant to grow normally, even at higher levels of metal in the medium [72]. In the current scenario, the accumulation of the metal increased with an increase in the exposure duration. Lower accumulation was noted in the sunflower with a duration of exposure to the metal of 15 days, followed by 30 days of exposure, and higher accumulation was recorded at 60 days of cadmium exposure. Similarly, higher translocation to the upper parts was also recorded. Higher absorption and accumulation were recorded by the roots and were subsequently translocated to the aerial parts of the plants. The accumulation of cadmium increased in the roots with the increase in metal supplements in the soil. The translocation and subsequent accumulation showed a decline in the case of the stem as the metal supplements increased in the soil [73]. The EDTA and IAA supplements increased the metal accumulation in the stem [68,74]. The highest translocation was recorded to the leaves of the sunflower, which showed a higher accumulation of cadmium. A positive correlation was recorded with the metal levels. Similarly, the rate of translocation and accumulation was higher with the application of both EDTA in the soil and foliar IAA [75]. For instance, no accumulation of cadmium was recorded in the seeds of the host plants.

## 5. Conclusions

The current evidence shows that Cd at higher concentrations actively accumulated in the hyperaccumulating plants, and beyond the threshold level (the WHO permissible level of 0.003 mg/kg), Cd exerted toxic effects in its host. The order of *H. annuus* plant parts based on the Cd concentration was stem > leaves > root and shoot > root. However, no accumulation or translocation were recorded in the seeds at any level of the metal and exposure time, ensuring food safety. The Cd accumulation in the leaves, stems, and roots increased in combination with EDTA and IAA compared to Cd applications alone in the control. The concentrations of Cd after 60 days in *H. annuus* subjected to Cd150 + EDTA + IAA exhibited a maximum accumulation of Cd of 64.80 mg/kg. The application of EDTA in soil and foliar IAA further improved the phytoremediation potential of the host and reduced the metal contaminant efficiently at the site. The application of these chemicals could be the possible solution for the rapid and enhanced bioremediation of sites contaminated with higher levels of cadmium.

## Data Availability

All the data are included in the manuscript.

## References

[1] Okereafor U., Makhatha M., Mekuto L., Uche-Okereafor N., Sebola T., Mavumengwana V. (2020). Toxic metal implications on agricultural soils, plants, animals, aquatic life and human health. Int. J. Environ. Res. Public Health.

[2] Rajendran S., Priya T., Khoo K.S., Hoang T.K., Ng H.-S., Munawaroh H.S.H., Karaman C., Orooji Y., Show P. (2022). A critical review on various remediation approaches for heavy metal contaminants removal from contaminated soils. Chemosphere.

[3] Mottet A., Tempio G. (2017). Global poultry production: Current state and future outlook and challenges. World’s Poult. Sci. J..

[4] Li C., Zhou K., Qin W., Tian C., Qi M., Yan X., Han W.J.S. (2019). A review on heavy metals contamination in soil: Effects, sources, and remediation techniques. Soil Sediment Contam. Int. J..

[5] Mohammed A.S., Kapri A., Goel R. (2011). Heavy metal pollution: Source, impact, and remedies. Biomanagement of Metal-Contaminated Soils.

[6] Duplay J., Semhi K., Errais E., Imfeld G., Babcsanyi I., Perrone T. (2014). Copper, zinc, lead and cadmium bioavailability and retention in vineyard soils (Rouffach, France): The impact of cultural practices. Geoderma.

[7] Degryse F., Shahbazi A., Verheyen L., Smolders E. (2012). Diffusion limitations in root uptake of cadmium and zinc, but not nickel, and resulting bias in the Michaelis constant. Plant Physiol..

[8] Shahid M., Dumat C., Khalid S., Niazi N.K., Antunes P. (2016). Cadmium bioavailability, uptake, toxicity and detoxification in soil-plant system. Rev. Environ. Contam. Toxicol..

[9] Jadia C.D., Fulekar M.H. (2009). Phytoremediation of heavy metals: Recent techniques. Afr. J. Biotechnol..

[10] Kalay M., Canli M. (2000). Elimination of essential (Cu, Zn) and non-essential (Cd, Pb) metals from tissues of a freshwater fish Tilapia zilli. Turk. J. Zool..

[11] Waseem A., Arshad J., Iqbal F., Sajjad A., Mehmood Z., Murtaza G. (2014). Pollution status of Pakistan: A retrospective review on heavy metal contamination of water, soil, and vegetables. BioMed Res. Int..

[12] Garbisu C., Alkorta I. (2001). Phytoextraction: A cost-effective plant-based technology for the removal of metals from the environment. Bioresour. Technol..

[13] Ashraf S., Ali Q., Zahir Z.A., Ashraf S., Asghar H.N. (2019). Phytoremediation: Environmentally sustainable way for reclamation of heavy metal polluted soils. Ecotoxicol. Environ. Saf..

[14] Yan A., Wang Y., Tan S.N., Mohd Yusof M.L., Ghosh S., Chen Z. (2020). Phytoremediation: A promising approach for revegetation of heavy metal-polluted land. Front. Plant Sci..

[15] Bai B., Nie Q., Zhang Y., Wang X., Hu W. (2021). Cotransport of heavy metals and SiO_2_ particles at different temperatures by seepage. J. Hydrol..

[16] Bai B., Jiang S., Liu L., Li X., Wu H. (2021). The transport of silica powders and lead ions under unsteady flow and variable injection concentrations. Powder Technol..

[17] Singh T.B., Ali A., Prasad M., Yadav A., Shrivastav P., Goyal D., Dantu P.K. (2020). Role of organic fertilizers in improving soil fertility. Contaminants in Agriculture.

[18] Hussain A., Hasnain S. (2011). Interactions of bacterial cytokinins and IAA in the rhizosphere may alter phytostimulatory efficiency of rhizobacteria. World J. Microbiol. Biotechnol..

[19] Warrier R., Paul M., Vineetha M. (2013). Estimation of salicylic acid in Eucalyptus leaves using spectrophotometric methods. Genet. Plant Physiol..

[20] El Far M., Taie H.A. (2009). Antioxidant activities, total anthocyanins, phenolics and flavonoids contents of some sweetpotato genotypes under stress of different concentrations of sucrose and sorbitol. Aust. J. Basic Appl. Sci..

[21] Bates L.S., Waldren R.P., Teare I. (1973). Rapid determination of free proline for water-stress studies. Plant Soil.

[22] Radhakrishnan R., Lee I.-J. (2013). Ameliorative effects of spermine against osmotic stress through antioxidants and abscisic acid changes in soybean pods and seeds. Acta Physiol. Plant..

[23] Asada K. (1992). Ascorbate peroxidase—A hydrogen peroxide-scavenging enzyme in plants. Physiol. Plant..

[24] Bradford M.M. (1976). A rapid and sensitive method for the quantitation of microgram quantities of protein utilizing the principle of protein-dye binding. Anal. Biochem..

[25] Schoefs B.t. (2002). Chlorophyll and carotenoid analysis in food products. Properties of the pigments and methods of analysis. Trends Food Sci. Technol..

[26] Hussain A., Alamzeb S., Begum S. (2013). Accumulation of heavy metals in edible parts of vegetables irrigated with waste water and their daily intake to adults and children, District Mardan, Pakistan. Food Chem..

[27] Kloppenburg J. (2018). Agriculturalists’ Reclamation. Ph.D. Thesis.

[28] Lyons G., Stangoulis J., Graham R. (2003). High-selenium wheat: Biofortification for better health. Nutr. Res. Rev..

[29] Bhargava A., Carmona F.F., Bhargava M., Srivastava S. (2012). Approaches for enhanced phytoextraction of heavy metals. J. Environ. Manag..

[30] Rahman Z., Singh V.P. (2019). The relative impact of toxic heavy metals (THMs)(arsenic (As), cadmium (Cd), chromium (Cr)(VI), mercury (Hg), and lead (Pb)) on the total environment: An overview. Environ. Monit. Assess..

[31] Kinuthia G.K., Ngure V., Beti D., Lugalia R., Wangila A., Kamau L. (2020). Levels of heavy metals in wastewater and soil samples from open drainage channels in Nairobi, Kenya: Community health implication. Sci. Rep..

[32] Liu L., Li W., Song W., Guo M. (2018). Remediation techniques for heavy metal-contaminated soils: Principles and applicability. Sci. Total Environ..

[33] Zafar-ul-Hye M., Naeem M., Danish S., Khan M.J., Fahad S., Datta R., Brtnicky M., Kintl A., Hussain G.S., El-Esawi M.A. (2020). Effect of cadmium-tolerant rhizobacteria on growth attributes and chlorophyll contents of bitter gourd under cadmium toxicity. Plants.

[34] Yang H., Shi G., Xu Q., Wang H. (2011). Cadmium effects on mineral nutrition and stress in Potamogeton crispus. Russ. J. Plant Physiol..

[35] Gill S.S., Khan N.A., Tuteja N. (2012). Cadmium at high dose perturbs growth, photosynthesis and nitrogen metabolism while at low dose it up regulates sulfur assimilation and antioxidant machinery in garden cress (*Lepidium sativum* L.). Plant Sci..

[36] Nada E., Ferjani B.A., Ali R., Bechir B.R., Imed M., Makki B. (2007). Cadmium-induced growth inhibition and alteration of biochemical parameters in almond seedlings grown in solution culture. Acta Physiol. Plant..

[37] Nabi A., Aftab T., Masroor M., Khan A., Naeem M. (2022). Exogenous triacontanol provides tolerance against arsenic-induced toxicity by scavenging ROS and improving morphology and physiological activities of *Mentha arvensis* L.. Environ. Pollut..

[38] Ma J., Saleem M.H., Alsafran M., Al Jabri H., Rizwan M., Nawaz M., Ali S., Usman K. (2022). Response of cauliflower (*Brassica oleracea* L.) to nitric oxide application under cadmium stress. Ecotoxicol. Environ. Saf..

[39] Muradoglu F., Gundogdu M., Ercisli S., Encu T., Balta F., Jaafar H.Z., Zia-Ul-Haq M. (2015). Cadmium toxicity affects chlorophyll a and b content, antioxidant enzyme activities and mineral nutrient accumulation in strawberry. Biol. Res..

[40] Govind G., Kulkarni J., Shinde H., Dudhate A., Srivastava A., Suprasanna P. (2022). Plant abiotic stress tolerance on the transcriptomics atlas. Advancements in Developing Abiotic Stress-Resilient Plants.

[41] Husna, Hussain A., Shah M., Hamayun M., Iqbal A., Qadir M., Alataway A., Dewidar A.Z., Elansary H.O., Lee I.-J. (2022). Phytohormones producing rhizobacteria alleviate heavy metals stress in soybean through multilayered response. Microbiol. Res..

[42] Salvi P., Manna M., Kaur H., Thakur T., Gandass N., Bhatt D., Muthamilarasan M. (2021). Phytohormone signaling and crosstalk in regulating drought stress response in plants. Plant Cell Rep..

[43] Ma X., Zhao X., Zhang Q., Zhou Z., Dou Y., Ji W., Li J. (2022). Comparative transcriptome analysis of broccoli seedlings under different Cd exposure levels revealed possible pathways involved in hormesis. Sci. Hortic..

[44] Fahad S., Hussain S., Bano A., Saud S., Hassan S., Shan D., Khan F.A., Khan F., Chen Y., Wu C. (2015). Potential role of phytohormones and plant growth-promoting rhizobacteria in abiotic stresses: Consequences for changing environment. Environ. Sci. Pollut. Res..

[45] Qadir M., Hussain A., Shah M., Lee I.J., Iqbal A., Irshad M., Sayyed A., Ahmad A., Hamayun M. (2022). Comparative assessment of chromate bioremediation potential of Pantoea conspicua and *Aspergillus niger*. J. Hazard. Mater..

[46] Yan Z., Tam N.F.Y. (2013). Effects of lead stress on anti-oxidative enzymes and stress-related hormones in seedlings of Excoecaria agallocha Linn. Plant Soil.

[47] Husna, Hussain A., Shah M., Hamayun M., Iqbal A., Murad W., Irshad M., Qadir M., Kim H.-Y. (2021). Pseudocitrobacter anthropi reduces heavy metal uptake and improves phytohormones and antioxidant system in *Glycine max* L.. World J. Microbiol. Biotechnol..

[48] Qadir M., Hussain A., Hamayun M., Shah M., Iqbal A., Irshad M., Ahmad A., Lodhi M.A., Lee I.-J. (2021). Phytohormones Producing Acinetobacter bouvetii P1 Mitigates Chromate Stress in Sunflower by Provoking Host Antioxidant Response. Antioxidants.

[49] Ibrahim M.H., Chee Kong Y., Mohd Zain N.A. (2017). Effect of Cadmium and Copper Exposure on Growth, Secondary Metabolites and Antioxidant Activity in the Medicinal Plant Sambung Nyawa (*Gynura procumbens* (Lour.) Merr). Molecules.

[50] Chen Q.Y., Murphy A., Sun H., Costa M. (2019). Molecular and epigenetic mechanisms of Cr (VI)-induced carcinogenesis. Toxicol. Appl. Pharmacol..

[51] Hamayun M., Khan N., Khan M.N., Qadir M., Hussain A., Iqbal A., Khan S.A., Rehman K.U., Lee I.-J. (2021). Antimicrobial and plant growth-promoting activities of bacterial endophytes isolated from Calotropis procera (Ait.) WT Aiton. Biocell.

[52] Waśkiewicz A., Muzolf-Panek M., Goliński P., Ahmad P., Azooz M.M., Prasad M.N.V. (2013). Phenolic content changes in plants under salt stress. Ecophysiology and Responses of Plants under Salt Stress.

[53] Mitra S., Pramanik K., Sarkar A., Ghosh P.K., Soren T., Maiti T.K. (2018). Bioaccumulation of cadmium by Enterobacter sp. and enhancement of rice seedling growth under cadmium stress. Ecotoxicol. Environ. Saf..

[54] Hamayun M., Hussain A., Iqbal A., Khan S.A., Lee I.-J. (2018). Endophytic fungus Aspergillus japonicus mediates host plant growth under normal and heat stress conditions. BioMed Res. Int..

[55] Ismail A.H., Mehmood A., Qadir M., Husna A.I., Hamayun M., Khan N. (2020). Thermal stress alleviating potential of endophytic fungus rhizopus oryzae inoculated to sunflower (*Helianthus annuus* L.) and soybean (*Glycine max* L.). Pak. J. Bot..

[56] Zhang S., Zhang H., Qin R., Jiang W., Liu D. (2009). Cadmium induction of lipid peroxidation and effects on root tip cells and antioxidant enzyme activities in *Vicia faba* L.. Ecotoxicology.

[57] Zulfiqar U., Ayub A., Hussain S., Waraich E.A., El-Esawi M.A., Ishfaq M., Ahmad M., Ali N., Maqsood M.F. (2022). Cadmium Toxicity in Plants: Recent Progress on Morpho-physiological Effects and Remediation Strategies. J. Soil Sci. Plant Nutr..

[58] Trchounian A., Petrosyan M., Sahakyan N., Gupta D.K., Palma J.M., Corpas F.J. (2016). Plant cell redox homeostasis and reactive oxygen species. Redox State as a Central Regulator of Plant-Cell Stress Responses.

[59] Saxena S.C., Salvi P., Kamble N.U., Joshi P.K., Majee M., Arora S. (2020). Ectopic overexpression of cytosolic ascorbate peroxidase gene (*Apx1*) improves salinity stress tolerance in *Brassica juncea* by strengthening antioxidative defense mechanism. Acta Physiol. Plant..

[60] Verma D., Upadhyay S.K., Singh K. (2022). Characterization of APX and APX-R gene family in Brassica juncea and B. rapa for tolerance against abiotic stresses. Plant Cell Rep..

[61] Upadhyay M.K., Majumdar A., Srivastava A.K., Bose S., Suprasanna P., Srivastava S. (2022). Antioxidant enzymes and transporter genes mediate arsenic stress reduction in rice (*Oryza sativa* L.) upon thiourea supplementation. Chemosphere.

[62] Ardestani M.M., van Gestel C.A.M. (2013). Using a toxicokinetics approach to explain the effect of soil pH on cadmium bioavailability to Folsomia candida. Environ. Pollut..

[63] Van Gestel C.A.M., van Diepen A.M.F. (1997). The Influence of Soil Moisture Content on the Bioavailability and Toxicity of Cadmium forFolsomia candidaWillem (Collembola: Isotomidae). Ecotoxicol. Environ. Saf..

[64] Almås Å., Singh B. (2001). Plant uptake of cadmium-109 and zinc-65 at different temperature and organic matter levels. J. Environ. Qual..

[65] Qadir M., Hussain A., Hamayun M., Shah M., Iqbal A., Husna, Murad W. (2020). Phytohormones producing rhizobacterium alleviates chromium toxicity in *Helianthus annuus* L. by reducing chromate uptake and strengthening antioxidant system. Chemosphere.

[66] Guo J., Tang S., Ju X., Ding Y., Liao S., Song N. (2011). Effects of inoculation of a plant growth promoting rhizobacterium *Burkholderia* sp. D54 on plant growth and metal uptake by a hyperaccumulator *Sedum alfredii* Hance grown on multiple metal contaminated soil. World J. Microbiol. Biotechnol..

[67] Zahoor M., Irshad M., Rahman H., Qasim M., Afridi S.G., Qadir M., Hussain A. (2017). Alleviation of heavy metal toxicity and phytostimulation of *Brassica campestris* L. by endophytic *Mucor* sp. MHR-7. Ecotoxicol. Environ. Saf..

[68] Seth C.S., Misra V., Singh R.R., Zolla L. (2011). EDTA-enhanced lead phytoremediation in sunflower (*Helianthus annuus* L.) hydroponic culture. Plant Soil.

[69] Sun S., Zhou X., Cui X., Liu C., Fan Y., McBride M.B., Li Y., Li Z., Zhuang P. (2020). Exogenous plant growth regulators improved phytoextraction efficiency by *Amaranths hypochondriacus* L. in cadmium contaminated soil. Plant Growth Regul..

[70] Husna, Hussain A., Shah M., Hamayun M., Qadir M., Iqbal A. (2022). Heavy metal tolerant endophytic fungi Aspergillus welwitschiae improves growth, ceasing metal uptake and strengthening antioxidant system in Glycine maxL. Environ. Sci. Pollut. Res..

[71] Zhang A., Yang X., Lu J., Song F., Sun J., Wang C., Lian J., Zhao L., Zhao B. (2021). OsIAA20, an Aux/IAA protein, mediates abiotic stress tolerance in rice through an ABA pathway. Plant Sci..

[72] Sánchez-Rodríguez C., Rubio-Somoza I., Sibout R., Persson S. (2010). Phytohormones and the cell wall in Arabidopsis during seedling growth. Trends Plant Sci..

[73] Fırat Ö., Çogun H.Y., Aslanyavrusu S., Kargın F. (2009). Antioxidant responses and metal accumulation in tissues of Nile tilapia Oreochromis niloticus under Zn, Cd and Zn + Cd exposures. J. Appl. Toxicol..

[74] Qadir M., Hussain A., Shah M., Hamayun M., Iqbal A. (2022). Enhancement of chromate phytoremediation and soil reclamation potential of *Brassica campestris* L. by *Aspergillus niger*. Environ. Sci. Pollut. Res..

[75] Hadi F., Bano A., Fuller M.P. (2010). The improved phytoextraction of lead (Pb) and the growth of maize (*Zea mays* L.): The role of plant growth regulators (GA3 and IAA) and EDTA alone and in combinations. Chemosphere.

